# Sol–gel auto combustion synthesis, characterization, and application of Tb_2_FeMnO_6_ nanostructures as an effective photocatalyst for the discoloration of organic dye contaminants in wastewater

**DOI:** 10.1039/d1ra02609g

**Published:** 2021-08-06

**Authors:** Mina Dara, Mohammad Hassanpour, Omid Amiri, Mahin Baladi, Masoud Salavati-Niasari

**Affiliations:** Institute of Nano Science and Nano Technology, University of Kashan Kashan P.O. Box 87317-51167 I. R. Iran Salavati@kashanu.ac.ir +98 31 5591 3201 +98 31 5591 2383; Faculty of Chemistry, Razi University Kermanshah 6714414971 Iran; Department of Chemistry, College of Science, University of Raparin Rania Kurdistan Region Iraq

## Abstract

In this study, the auto-combustion sol–gel method was used to prepare novel Tb_2_FeMnO_6_ (TFMO) double perovskite nanoparticles. Chemical and natural fuels were used to achieve these particles with appropriate size. The resulting particles were examined *via* X-ray powder diffraction (XRD) and scanning electron microscopy (SEM) techniques. Rietveld analysis was also performed to confirm the crystallinity and lattice parameters of the formed particles. The particles obtained in the presence of maleic acid were selected as the optimal sample (S4), and the particles obtained in the presence of pomegranate paste were chosen as the non-optimal sample (S8) in terms of size and morphology. Both particles were used to investigate the photocatalytic activity. Fourier transform infrared spectroscopy (FTIR), UV-Vis diffuse reflectance spectroscopy (DRS), and vibrating sample magnetometer (VSM) analyses and N_2_ adsorption/desorption isotherms were performed for both samples and the results were compared. Erythrosine and malachite green dyes in aqueous solutions were used as contaminants in the photocatalysis process. The results showed 22% and 20% discoloration for S4 and 41% and 30% discoloration for S8 in the presence of erythrosine and malachite green under visible light irradiation. The photocatalytic activity was investigated under UV light for S4, which showed 80% and 50% discoloration for erythrosine and malachite green, respectively. Investigating the photocatalytic activity of TFMO double perovskite nanoparticles showed that these nanoparticles could be a desirable option for mitigating water pollution.

## Abstract 2

In this study, new double perovskite Tb_2_FeMnO_6_ nanoparticles were successfully synthesized by a sol–gel auto combustion method for the first time. The photocatalytic properties of the nanoparticles were also evaluated for the first time.

## Graphical abstract

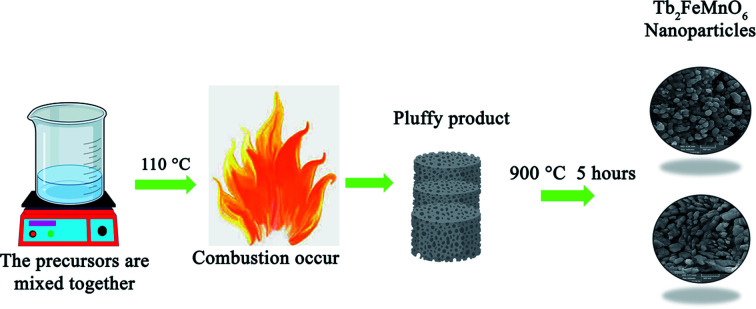

## 1. Introduction

Potable water is one of the oldest problems that humans have been trying to solve for a long time.^1^ Due to the scarcity of water resources and some environmental problems related to wastewater, wastewater treatment and industrial effluents are of particular importance in this area. Numerous methods have been used for wastewater treatment, such as biological methods, coagulation, adsorption by activated carbon, ion exchange, electrochemical, and photocatalytic processes.^2–7^

Photocatalysis is a process that uses light radiation to create electron–holes. The reaction of these electron–holes with one of the species in the solution produces free radicals, which are eventually used to remove contaminants.^8^ Among the widely used materials in the photocatalysis process, materials with semiconductor properties are worth mentioning.^9^ This property can be improved through nanotechnology. Therefore, nanoscale semiconductors are known as the leading candidates for the photocatalytic process.^10^ Therefore, the development of nanoparticles and introduction of new structures to improve and advance the photocatalytic process have been of interest to researchers in recent years.

The family of perovskites with the structure ABO_3_ is considered as one of the attractive categories of materials due to their interesting physical properties and application in various devices such as sensors, supercapacitors, and catalysts.^11–13^ Double perovskites belonging to this group are formed by occupying the B-site with two different cations.^14^ Due to their unique properties, many applications of double perovskites such as in photovoltaics, optoelectronics, catalysis, solar-cells, and photocatalysis have been mentioned in literature.^15–18^ The use of rare-earth elements at A-site in the structure A_2_BBO_6_ has been reported in the literature. Soofivand *et al.* synthesized Dy_2_ZnMnO_6_ nanoparticles by a sonochemical method, where the nanoparticles were obtained after calcination at 900 °C. They used Dy_2_ZnMnO_6_ double perovskite to remove the dye from an aqueous solution containing methyl violet.^19^ In another study, Gd_2_NiMnO_6_ nanostructures were synthesized in the presence of different saccharides by combustion method wherein the electrochemical, optical, and magnetic behaviors of rare-earth double perovskites were investigated.^20^

Based on the numerous reports on the synthesis of double perovskites and the appropriate efficiencies of these materials in the photocatalytic process, our team decided to synthesize new nanoparticles from this class of materials.

In literature, the conventional method for preparing these particles seems to be the solid-state synthesis. For example, Tb_2_CoMnO_6_ particles were synthesized by the conventional solid-state reaction, and their magnetic properties were investigated.^21^ Also, Tb_2_NiMnO_6_ particles were prepared by the solid-state method with Tb_2_O_3_, NiO, and MnO_2_ metal oxides, and their dielectric properties were investigated.^22^ In this study, Tb_2_FeMnO_6_ nanoparticles were synthesized by a sol–gel auto-combustion method for the first time. The performed analyses confirmed the double perovskite structure. Due to the diverse and specific properties mentioned for these structures, the ability of these synthesized particles as a candidate in the photocatalysis process was investigated for the first time. For a better investigation, two samples with different sizes were compared in a photocatalytic process to remove colored contaminants.

## 2. Experimental

### 2.1. Materials and characterization

All of the chemicals used in this study were of high-grade bought from Merck. A diffractometer of the Philips company with X'PertPro monochromatized Cu Kα radiation (*λ* = 1.54 Å) was used to collect XRD (X-ray diffraction) patterns to confirm the type of structure and the purity of the as-synthesized nanoparticles. The morphology and distribution of nanoparticles were investigated by FESEM (field emission scanning electron microscopy) (Mira3 tescan). The nanoparticles were examined *via* transmission electron microscopy on a JEM-2100 TEM. For EDS (energy dispersive spectrometry) analysis, a Philips XL30 microscope was used. A magnetometer device made by Meghnatis Daghigh Kavir Company from Iran was used to investigate the magnetic properties of the as-synthesized nanoparticles. Brunauer–Emmett–Teller (BET) method was used to determine the specific surface areas of the catalysts. The measurements were carried out at −196 °C by injecting liquid N_2_ to determine the adsorption/desorption using an automated gas adsorption analyzer (Tristar 3000, Micromeritics).

### 2.2. Prepared TFMO double perovskites

For the synthesis of TFMO by the sol–gel auto combustion method, 2 mmol of Tb(NO_3_)_3_·6H_2_O, 1 mmol of Fe(NO_3_)_3_·9H_2_O, and 1 mmol of Mn(NO_3_)_2_·6H_2_O were dissolved in distilled water separately to achieve a transparent solution. 1 mmol oxalic acid as the fuel was added to the Tb solution. Also, 1 mmol oxalic acid as the fuel was added to the solution containing the Tb precursor. After stirring for 5 min, the solution, including Fe and Mn, was added to the previous solution. In this step, the solution was mixed well for 30 min with a magnetic stirrer. After that, the reaction temperature was raised to 110 °C in order for the combustion process to take place. The resulting powder was transferred to a furnace and calcined at 700 °C for 5 h. The type and amount of the fuel and calcination temperature were changed to investigate the effects of different parameters on the synthesis of these particles. The different conditions used to synthesize these particles are presented in Table 1.

**Table 1:** Different reaction conditions for the preparation of TFMO nanoparticles

No.	Mole ratio of Tb : Fe : Mn	Fuel	Ratio of fuel to Tb	Calcination temperature (°C)	Time of calcination (h)
1	2 : 1 : 1	Oxalic acid	1 : 2	900	5
2	2 : 1 : 1	Oxalic acid	1 : 2	800	5
3	2 : 1 : 1	Oxalic acid	1 : 2	700	5
4	2 : 1 : 1	Maleic acid	1 : 2	700	5
5	2 : 1 : 1	Green coffee powder	1 g	700	5
6	2 : 1 : 1	Pomegranate paste	1 g	700	5
7	2 : 1 : 1	Pomegranate paste	2 g	700	5
8	2 : 1 : 1	Pomegranate paste	3 g	700	5

### 2.3. Photocatalysis path

0.05 g of the optimal samples was added to 100 ml of the dye solution at a concentration of 20 ppm to perform the photocatalysis process. At the beginning of the photocatalysis process, the solution was first aerated for 20 min. After aeration, the first sample was taken and irradiated with visible or UV light. Sampling was performed at 10 min intervals; however, initial sampling was performed after 20 min. All steps of the photocatalysis process were performed at ambient temperature and without external light irradiation. The discoloration of the dyes was checked by recording the absorbance using a UV-Visible spectrophotometer. The following formula was used to calculate the discoloration percentage (eqn (1)):

1 % Discoloration = (*A*_0_ − *A*_*t*_)/*A*_0_ × 100,

where *A*_0_ and *A*_*t*_ are the absorbance values of the dye solution at 0 and *t* min, respectively.^23^

## 3. Result and discussion

**Fig. 1:**
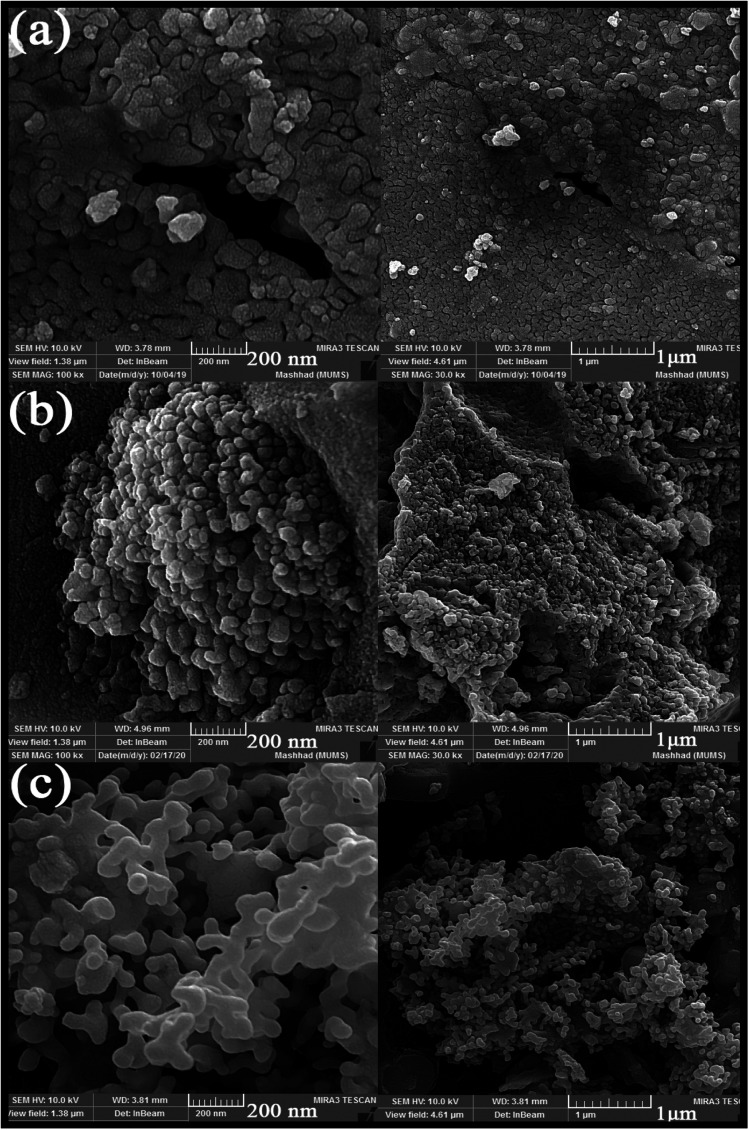
SEM images obtained from nanoparticles synthesized in the presence of different fuels: (a) oxalic acid S3, (b) maleic acid S4, and (c) green coffee powder S5.

Different fuels were used in the synthesis of double perovskite TFMO particles to obtain a suitable size and morphology. The ability of fuels to create complexes, reduction capability, and gas produced affects the morphology, purity, and porosity of the product.^24,25^ The presence of the fuel causes the solution to homogenize by forming a complex with metal ions. Furthermore, creating a complex plays a role, such as being the capping agent, and prevents the formation of by-products. Also, the type of the fuel used can be related to the intensity of combustion, which causes proper combustion and higher purity of the product. The SEM images were analysed to investigate the effects of fuels on the size and morphology of TFMO particles. Fig. 1(a)–(c) shows the SEM image of TFMO particles S3–S5. It can be seen that when oxalic acid was used as the fuel (Fig. 1(a)), particles adhered together, and the size of particles was coarser than the other samples. When the particles were obtained from maleic acid and green coffee powder as fuels was seen (Fig. 1(b) and (c)), it was determined that maleic acid had greater efficacy in the size control property compared to others.

**Fig. 2:**
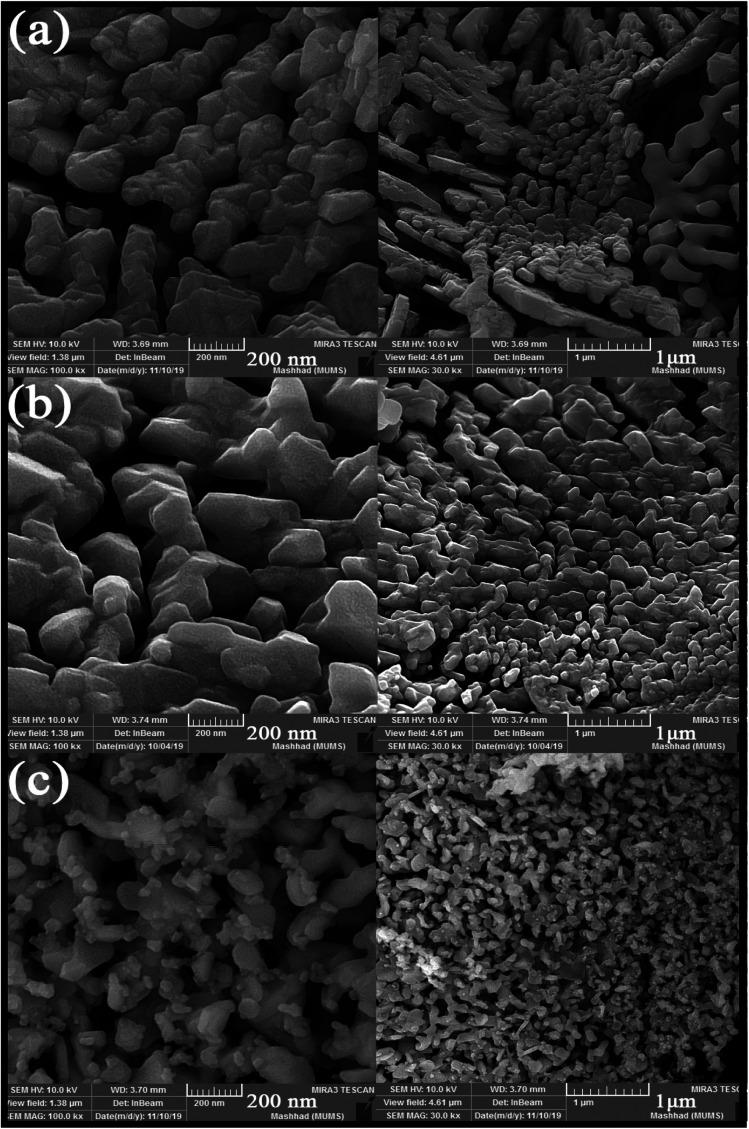
SEM images obtained from nanoparticles synthesized in the presence of different amounts of pomegranate paste as a fuel: (a) 1 g, (b) 2 g, and (c) 3 g.

SEM images obtained from adding pomegranate paste to the reaction medium as a natural fuel is shown in Fig. 2(a)–(c). Fig. 2 shows that by adding pomegranate paste as the fuel, particles with larger sizes are formed in comparison to the previous samples. One of the reasons for this could be that although natural materials act as the fuel needed to perform the reaction, the combustion operation remains incomplete due to the presence of other compounds in these materials. With the increase in the amount of pomegranate paste, it was expected that the particle size would become smaller, but there was not much change observed in the images.

**Fig. 3:**
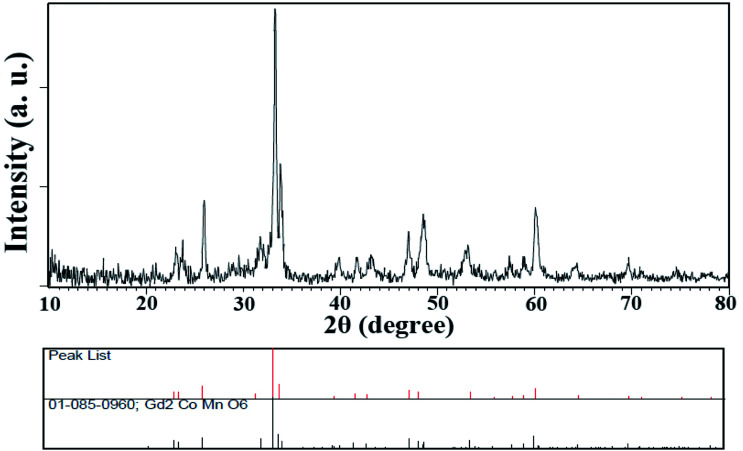
XRD pattern obtained from TFMO nanoparticles (S4).

**Fig. 4:**
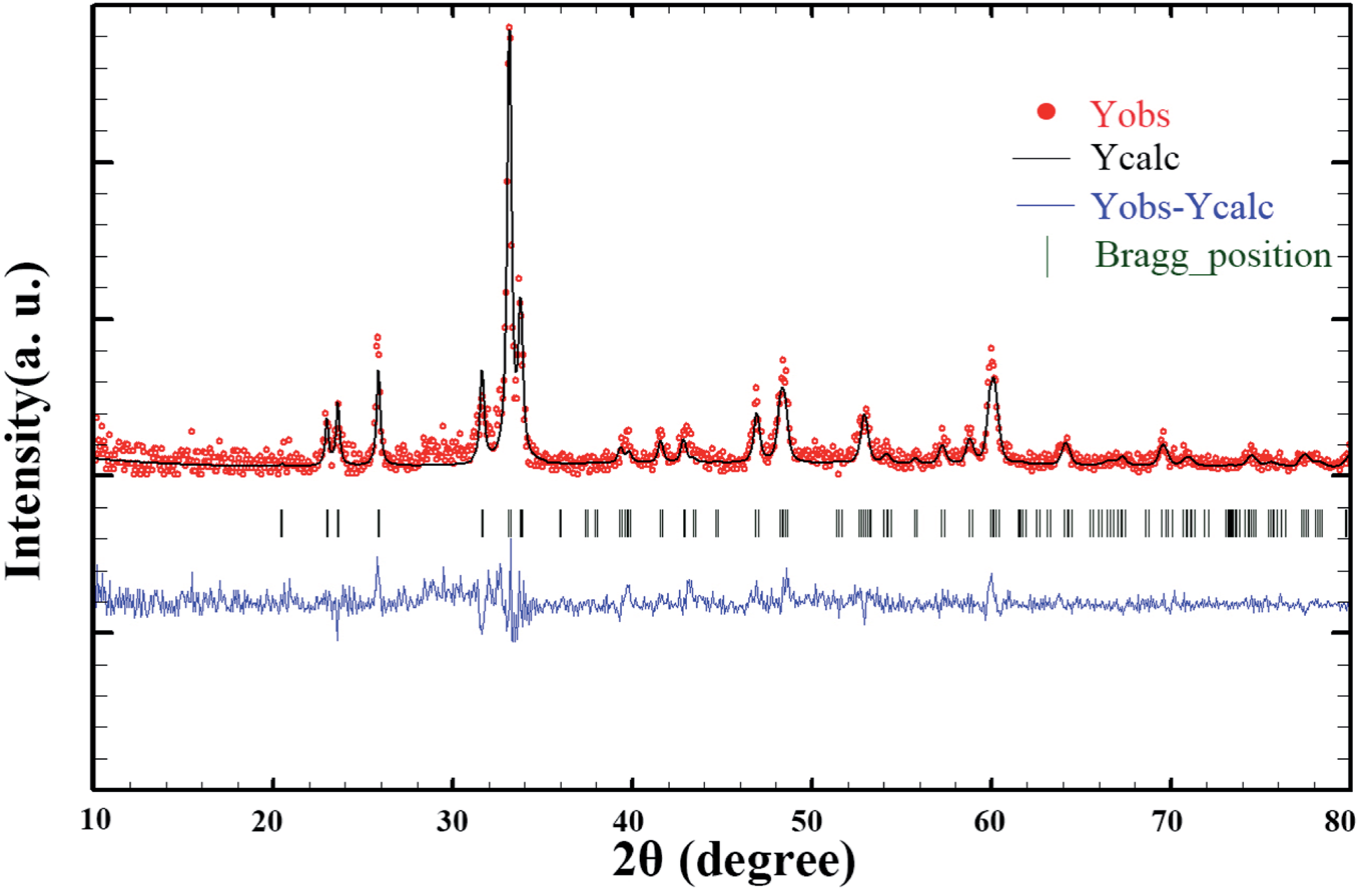
X-ray diffraction Rietveld refinement patterns of TFMO nanoparticles (S4).

**Fig. 5:**
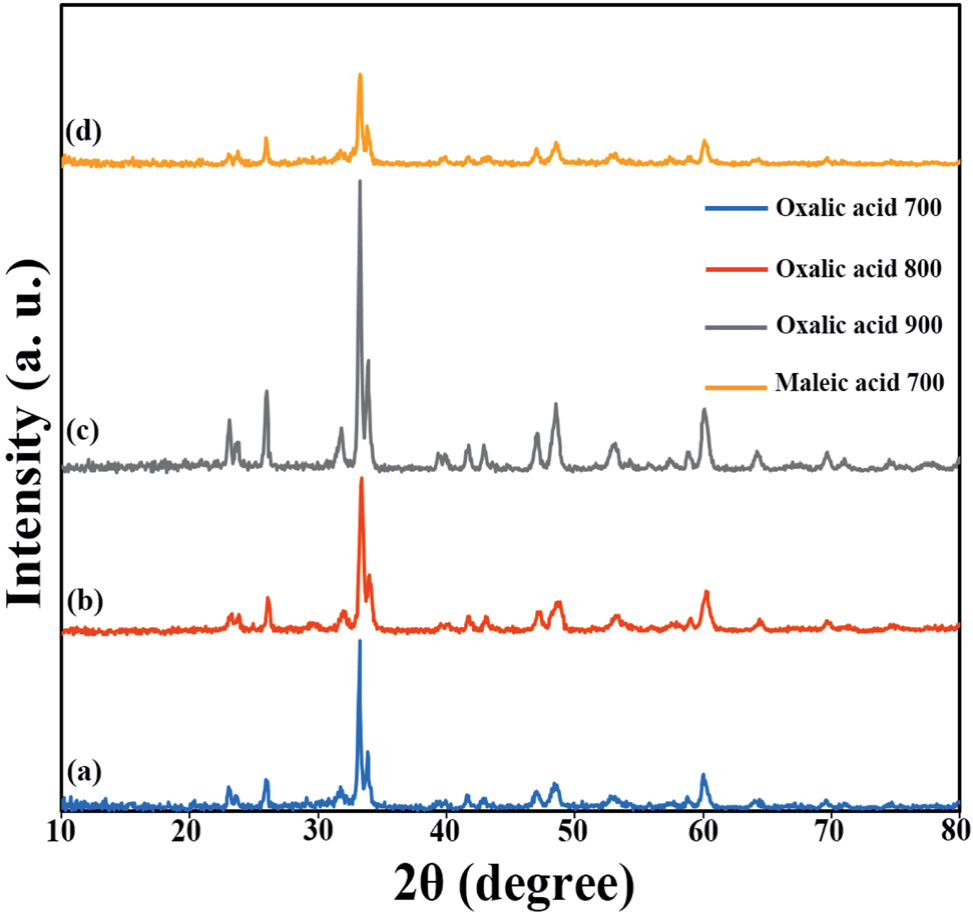
XRD patterns obtained from samples prepared in the presence of different fuels and at different calcination temperatures: (a) oxalic acid at 700 °C, (b) oxalic acid at 800 °C, (c) oxalic acid at 900 °C, and (d) maleic acid at 700 °C.

**Fig. 6:**
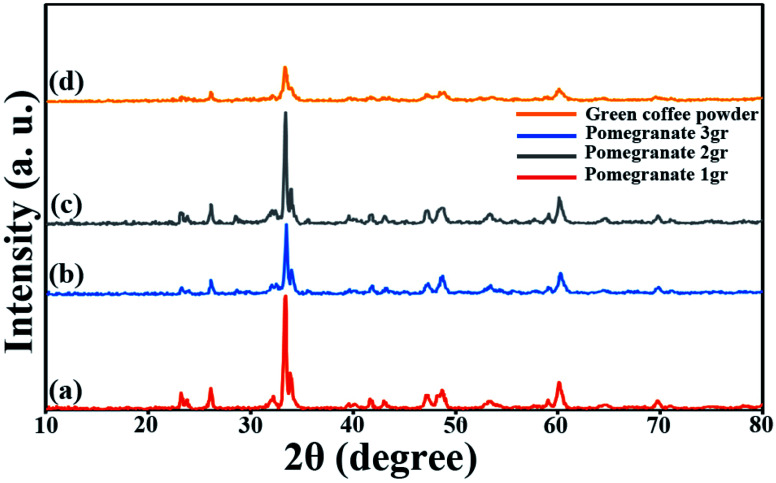
XRD patterns obtained from samples prepared in the presence of pomegranate paste in different amounts (a) 1 g, (b) 2 g, (c) 3 g, and (d) green coffee powder as a fuel after calcination at 700 °C.

Since these nanoparticles were synthesized for the first time, the XRD pattern recorded in the databases could not found. Hence, the XRD patterns obtained were compared with the existing patterns of other nanoparticles. A Gd_2_CoMnO_6_ pattern (JCPDS card number: 01-085-0960) was used to compare with the pattern obtained from this nanoparticle (Fig. 3). Comparison of peaks showed that double perovskite structure was formed. The data obtained by the X-ray analysis were used to accomplish the Rietveld structural refinement to obtain the structural parameters using the FULL PROF program.^26^ TbFeO_3_ structural data were used to perform the Rietveld analysis, which was done by considering the small difference in the ionic radii of Mn^3+^ (0.58 Å) and Fe^3+^ (0.55 Å). Also, the occupation of half of the Fe atom positions with Mn to achieve the TFMO structure was considered. The result of refined structural parameters in the orthorhombic space group *Pbnm* were *a* = 5.312, *b* = 5.659, *c* = 7.546 and the reliability factors *R*_p_ = 48.8, *R*_wp_ = 53.5, *R*_exp_ = 38.53 and *χ*^2^ = 1.93. The X-ray diffraction Rietveld refinement pattern is shown in Fig. 4. The XRD analysis was performed for all samples at room temperature. The obtained results are shown in Fig. 5 and 6, wherein different fuels and different calcination temperatures were applied. The Williamson–Hall equation can determine the effects of the lattice strain and the crystallite size on the FWHM value (eqn (2)):^27^2
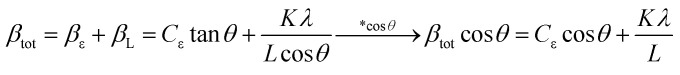
where *θ* is the Bragg's angle, *λ* is the wavelength of X-ray, *β*_tot_ is the peak width at half-maximum intensity, and *L* is the crystallite size. By plotting (*β*_tot_ cos *θ*) diagrams in (cos *θ*), the average size of the crystallite (*L*) can be calculated from the *y*-intercept. The crystallite size for samples S1–S8 was calculated about 26, 24, 23, 26, 24.5, 26, 25, and 31 nm, respectively.

**Fig. 7:**
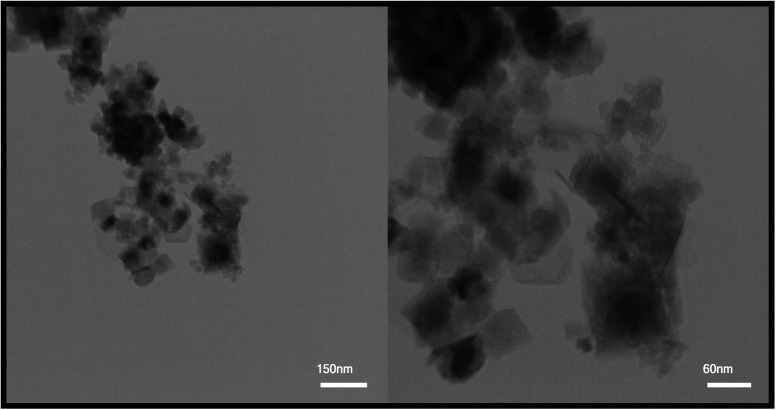
TEM images obtained for TFMO nanoparticles (S4).

After reviewing the images obtained from SEM and the data obtained from XRD, two samples were selected to continue the research. Sample S4, in which maleic acid was used as the fuel, was selected as the optimal sample to be compared with other samples due to its appropriate size and morphology. Also, to evaluate and compare two different particle sizes, the S8 sample with a larger particle size than the optimal sample and agglomerated particles was selected as the second sample. TEM images were used to further investigate the morphology of the particles. TEM images obtained from the TFMO nanoparticles of sample S4 are shown in Fig. 7. The nanoparticles obtained in the resulting images showed that the nanoparticles adhered together and agglomerated.

**Fig. 8:**
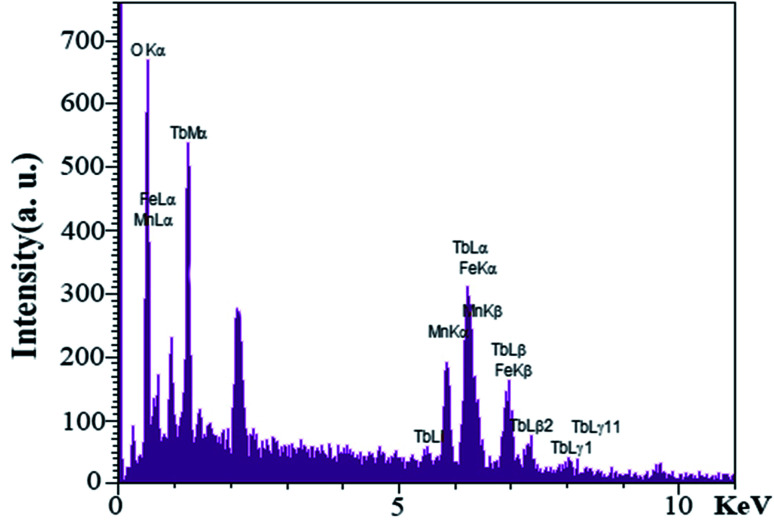
EDS spectra obtained for TFMO nanoparticles (S4).

EDS analysis was performed to evaluate the presence of elements and purity of the as-synthesized nanoparticles for S4. As shown in Fig. 8, an examination of the resulting spectrum confirmed the presence of the terbium, iron, manganese, and oxygen elements.

**Fig. 9:**
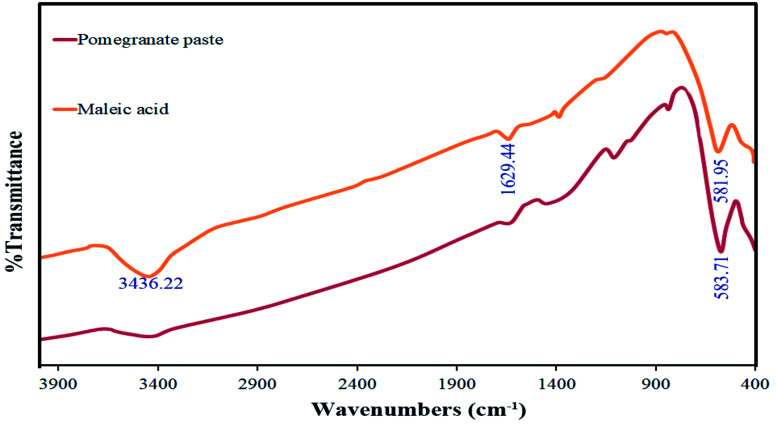
FT-IR spectrum obtained for TFMO nanoparticles in the presence of maleic acid (S4) and pomegranate paste (S8) as fuels.

FT-IR analysis was used to investigate the functional groups of the synthesized materials. The spectra of this analysis are shown in Fig. 9 for the two samples in which maleic acid (S4) and pomegranate paste (S8) were used as fuels. The bands observed at around 3400 cm^−1^ and 1620 cm^−1^, which are common to both spectra, are related to moisture absorption at the surface of the TFMO particles. For the sample prepared with pomegranate paste as a fuel, a minimal peak at around 1111 cm^−1^ can be seen, which can be attributed to organic compounds that entered the composition through pomegranate paste and were not entirely destroyed by the calcination process. The absorption bands at about 580 cm^−1^ are assigned to metal–oxygen bonds.^28^

**Fig. 10:**
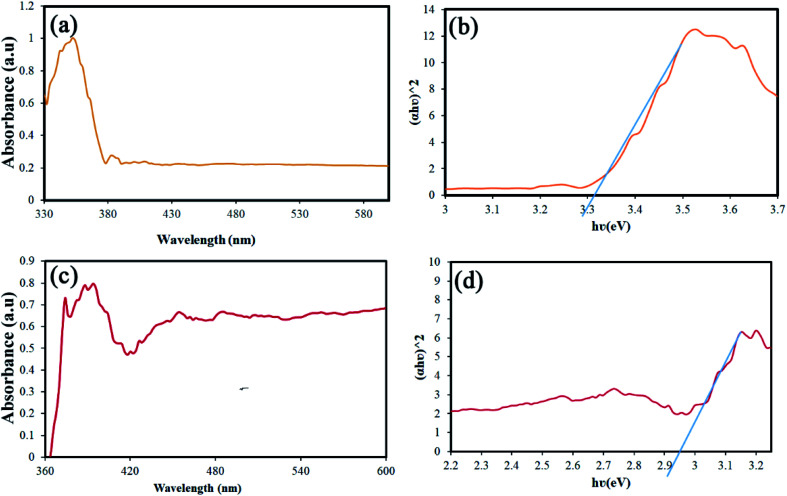
(a and c) Results of the DRS analysis, and (b and d) calculated the band-gap using the Tauc's equation of S4 and S8, respectively.

Band-gap is one of the parameters that can be changed and engineered according to particle size.^29,30^ The value of this parameter is also significant in performing the photocatalysis process. Accordingly, the DRS analysis was performed for two different sizes of samples. Fig. 10(a) and (c) shows the results of this analysis for S4 and S8. The Tauc's equation was used to calculate the band-gap from the data obtained.^31^ The band-gap values were calculated to be approximately 3.3 and 2.9 eV, respectively (Fig. 10(b) and (d)). The values obtained for the band-gap are in complete agreement with the size of the nanoparticles, which is quite visible in the SEM images.^32^

**Fig. 11:**
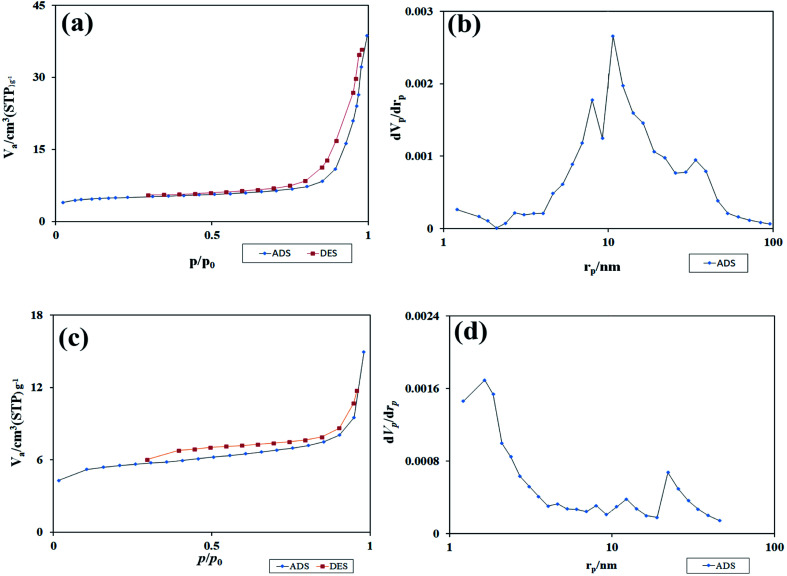
(a and c) The isotherm obtained from the study of N_2_ adsorption/desorption on TFMO particles, and (b and d) the graph obtained from BJH calculations of S4, and S8 respectively.

Fig. 11(a) and (c) shows the isotherm obtained from the study of N_2_ adsorption/desorption on TFMO particles. As it turns out, the isotherms formed for both S4 and S8 are type III isotherms with a type H3 hysteresis loop.^33^ Using the graph obtained from BJH calculations, the pore size distribution for the two samples S4 and S8 is shown in Fig. 11(b) and (d). The values obtained for the pore size distribution were about 10 nm in S4 and about 1.5 nm in S8 particles. These values indicate that the volume of pores in TFMO particles increases with the decrease in the particle size. The specific surface area and total pore volume of TFMO particles were measured by the BET method, which were about 18.231 m^2^ g^−1^ and 0.056 cm^3^ g^−1^ for S4, and about 20.28 m^2^ g^−1^ and 0.023 cm^3^ g^−1^ for S8, respectively. This analysis can provide helpful insight in examining the results obtained from the photocatalytic activity of nanoparticles.

**Fig. 12:**
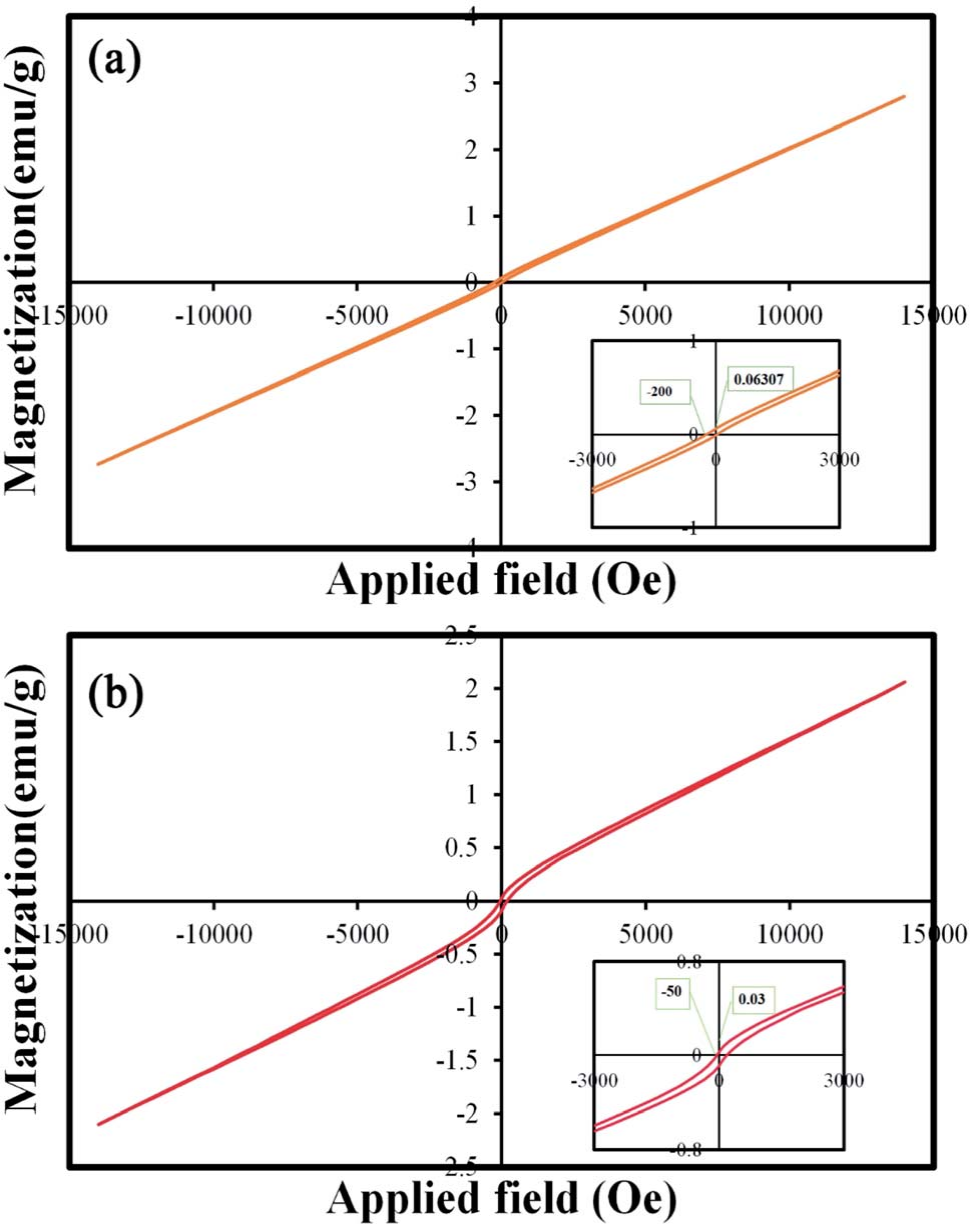
Magnetization *versus* applied magnetic field at room temperature and the inset shows the magnified hysteresis loop of (a) S4, and (b) S8.

One of the important properties of catalysts is their easy collection after the photocatalysis process. Catalysts with proper magnetic properties can be retrieved and reused easily.^34^ Therefore, the magnetic properties of these nanoparticles were investigated and reported. Fig. 12(a) and (b) show the *M*–*H* curves of TFMO particles S4 and S8, respectively. The hysteresis loops with coercive fields of 200 and 50 Oe and remnant magnetization of 0.06 and 0.03 emu g^−1^ for S4 and S8 were obtained, respectively. The results of the VSM analysis showed that both samples exhibited paramagnetic behavior. The increase in the loop volume for S8 can be attributed to the increase in the particle size of this sample.^35^

**Fig. 13:**
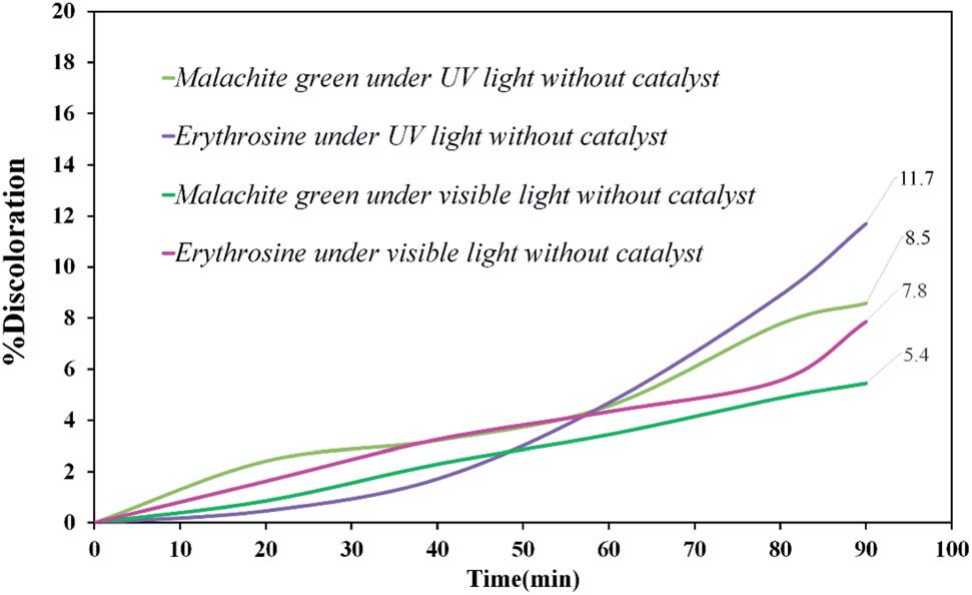
Discoloration of erythrosine (11.7%), malachite green (8.5%) under UV, and erythrosine (7.8%), malachite green (5.4%) under visible light.

**Fig. 14:**
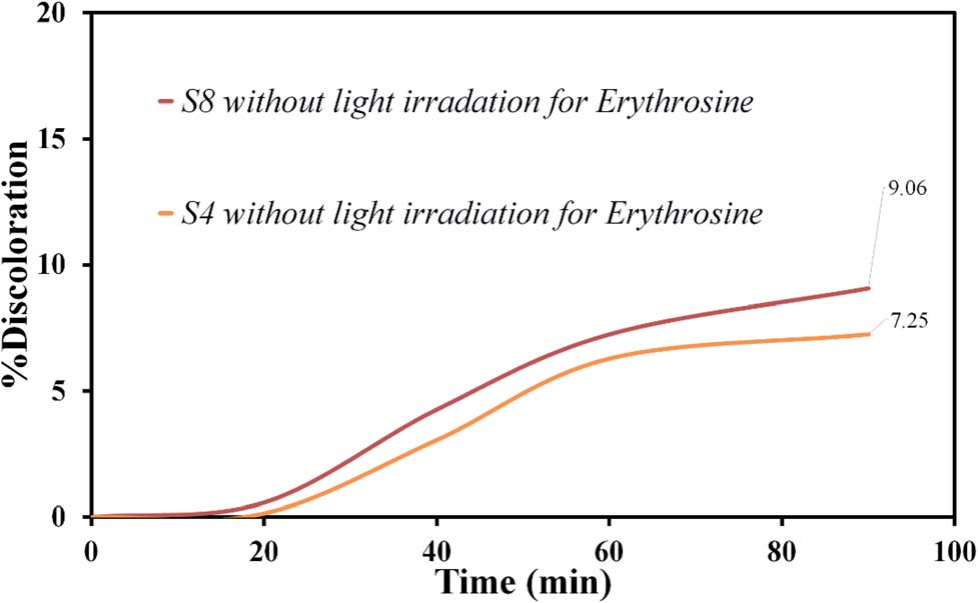
The experiment in dark to check for the adsorption of the dyes on the catalysts S4 and S8.

**Fig. 15:**
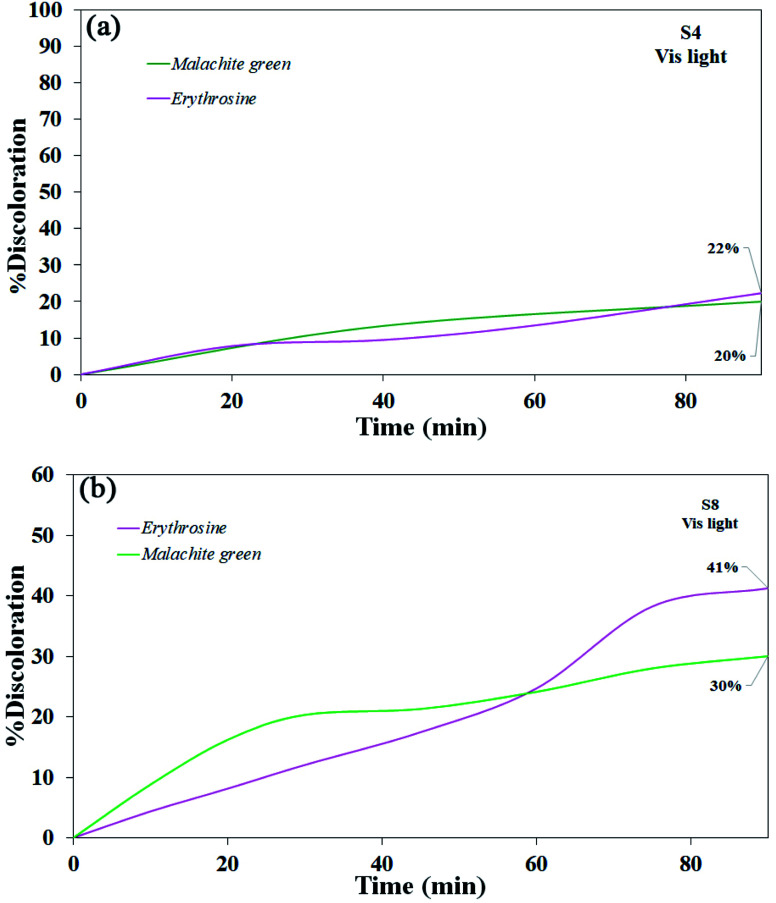
Photocatalytic activity of TFMO nanoparticles under visible light (a) S4 on discoloration erythrosine (22%) and malachite green (20%), and (b) S8 on discoloration erythrosine (41%) and malachite green (30%).

**Fig. 16:**
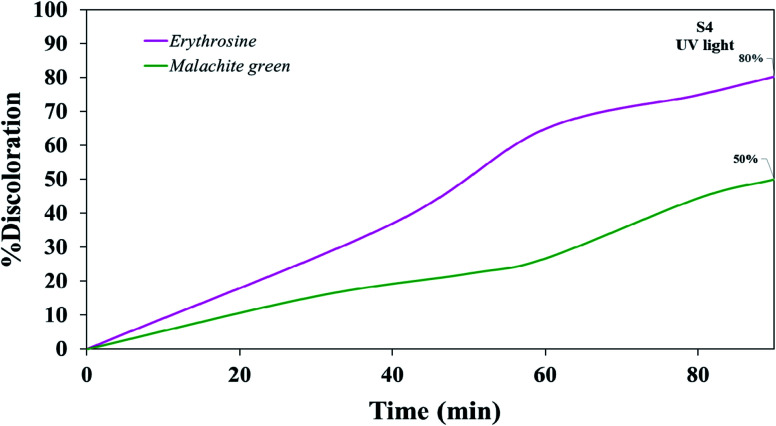
Photocatalytic activity of TFMO nanoparticles S4 on discoloration erythrosine (80%) and malachite green (50%) under UV light.

Various steps are required to accurately investigate the photocatalysis process, including the effect of light, the effect of dye adsorption on the catalyst, the pH and temperature effect, the effect of the photocatalyst, and so on.^23,36^ First, a control test was performed to investigate the effect of UV and visible light irradiation on the degradation of erythrosine and malachite green for 90 min at ambient temperature to study the effect of light alone, *i.e.*, photolysis. As shown in Fig. 13, both dyes were slightly degraded under visible and UV light. After that, the experiment was performed in dark to check for the adsorption of the dyes on the catalysts. This process was performed to evaluate the amount of dye adsorption on TFMO nanoparticles. The results showed that in about 90 min, only about 7% of discoloration was observed for S4 and about 9% for S8 (Fig. 14). In fact, this value indicates the adsorption of dye on the surface of the catalyst. In the following, the photocatalysis process was performed to remove erythrosine and malachite green from the aqueous solution under visible light. As shown in Fig. 15(a), discoloration in the presence of S4 as a catalyst for erythrosine and malachite green was obtained, 22% and 20%, respectively. As observed in the previous process, another process was performed in the presence of S8 as a catalyst, which showed discoloration of about 41% and 30% for erythrosine and malachite green, respectively (Fig. 15(b)). Observation of these results was predicted according to the surface and optical properties obtained from the samples. The obtained results showed the considerable role of dye adsorption on the catalyst and light irradiation to dye degradation under visible light. It was specified that the S4 catalyst has a negligible effect on the discoloration under visible light. Of course, it was predicted that due to the band-gap of about 3.3 eV calculated for S4, visible light with a wavelength of about 420 nm could not create electron–holes.^37^ In the following, the photocatalysis process was performed to investigate the optimal sample (S4) photocatalytic activity under UV light. According to Fig. 16, the photocatalysis test results for S4 showed 80% discoloration for erythrosine and 50% for malachite green. According to the obtained results, TFMO nanoparticles can be a suitable candidate for the photocatalytic process. However, it is possible to achieve better performance with changes in the process as well as changes in the size and morphology of these particles.

## 4. Conclusion

Briefly, in this study, TFMO nanoparticles were synthesized by the auto-combustion sol–gel method. Different fuels were used to investigate the effect of the fuel type on the particle size and morphology. Different analyses were performed to confirm the formation and purity of nanoparticles and study the optical and magnetic properties. Finally, the resulting nanoparticles in the presence of maleic acid as a fuel were selected as the optimal sample. The photocatalysis process was performed for two samples, S4 (maleic acid fuel) and S8 (pomegranate paste fuel), to investigate the photocatalytic activity of these nanoparticles and compare the photocatalysis of two size particles. Two control tests were performed for the photocatalysis process. First, dye degradation in the photolysis process under visible and UV light was performed to investigate the effects of light radiation on degradation. Then, to investigate the absorption of dye on the catalyst surface, the previous process was performed in the presence of a catalyst and without light. The control tests showed that both the photolysis and dye adsorption on the catalyst surface would play a small role in the final photocatalytic activity. For sample S4, with a band-gap of 3.3 eV, no significant discoloration was observed under visible light. However, discoloration was obtained at about 41% and 30% to remove erythrosine and green malachite for S8, respectively. The photocatalysis process was also performed for the optimal sample under UV light. The discoloration results showed 80% for erythrosine and 50% for malachite green. These results suggest TFMO double perovskite nanoparticles as a suitable candidate for the photocatalysis process. However, with changes in the synthesis conditions and obtaining particles with a more appropriate size and morphology, better performance in the photocatalysis process can be recorded.

## Conflicts of interest

The authors declare that there are no conflicts of interest regarding the publication of this manuscript.

## Supplementary Material
